# Evolutionary Relationships of Wild Hominids Recapitulated by Gut Microbial Communities

**DOI:** 10.1371/journal.pbio.1000546

**Published:** 2010-11-16

**Authors:** Howard Ochman, Michael Worobey, Chih-Horng Kuo, Jean-Bosco N. Ndjango, Martine Peeters, Beatrice H. Hahn, Philip Hugenholtz

**Affiliations:** 1 Department of Ecology and Evolutionary Biology, University of Arizona, Tucson, Arizona, United States of America; 2 Institute of Plant and Microbial Biology, Academia Sinica, Taipei, Taiwan; 3 Faculties of Sciences, University of Kisangani, Kisangani, Democratic Republic of the Congo; 4 Institut de Recherche pour le Développement (IRD), University of Montpellier 1, Montpellier, France; 5 Department of Medicine, University of Alabama at Birmingham, Birmingham, Alabama United States of America; 6 Department of Microbiology, University of Alabama at Birmingham, Birmingham, Alabama United States of America; 7 Microbial Ecology Program, DOE Joint Genome Institute, Walnut Creek, California, United States of America; Environmental Research Institute, University College Cork, Ireland

## Abstract

Multiple factors over the lifetime of an individual, including diet, geography, and physiologic state, will influence the microbial communities within the primate gut. To determine the source of variation in the composition of the microbiota within and among species, we investigated the distal gut microbial communities harbored by great apes, as present in fecal samples recovered within their native ranges. We found that the branching order of host-species phylogenies based on the composition of these microbial communities is completely congruent with the known relationships of the hosts. Although the gut is initially and continuously seeded by bacteria that are acquired from external sources, we establish that over evolutionary timescales, the composition of the gut microbiota among great ape species is phylogenetically conserved and has diverged in a manner consistent with vertical inheritance.

## Abstract 2

Although bacteria are continually acquired over the lifetime of an individual, the phylogenetic relationships of great ape species is mirrored in the compositions of their gut microbial communities.

## Author Summary

The microbial communities that inhabit the gastrointestinal tract of humans and other mammals are complex, dynamic, and critical to both health and disease. The composition and constituents of these communities are influenced by multiple factors such as host diet, geography, physiology, and disease state. Given the central role of the gut microbiota in the physiology of the host, it is important to determine whether it is predictable and substantially determined by the host, or variable and largely determined by the external environment (including diet) experienced by the host. A valuable way of determining the relative contributions of such factors is by comparing gut microbial communities in closely related host species. Applying a high-throughput sequencing approach, we profiled the distal gut microbiotae of great ape species sampled in their native ranges and then employed a parsimony-based analysis of phylogenetically informative phylotypes (i.e., bacterial taxa residing in multiple individuals) to determine the relationships among the diverse microbial communities. Our analyses revealed a clear species-specific signature of microbial community structure. Moreover, the pattern of relationships among the five great ape species (*Homo sapiens, Pan troglodytes, P. paniscus, Gorilla gorilla,* and *G. beringei*) inferred from their fecal microbial communities was identical to that inferred from host mitochondrial DNA, indicating that host phylogeny shapes the gut microbiota over evolutionary timescales. It seems after all that you are not what you eat.

## Introduction

The mammalian digestive tract is sterile at birth but is soon colonized by bacteria that typically derive from the mother [1]–[3]. In the absence of any subsequent alterations or additional colonizations, strict parental inheritance would result in a pattern in which the constituents and composition of the microbial flora would co-diversify with and ultimately mirror the evolutionary relationships of their hosts. Such a situation has been observed in some bacteria within the digestive tract, such as *Helicobacter pylori,* which is present in the stomachs of about half of the human population and whose patterns of divergence closely follow those of their human hosts [4].

Numerous internal and external factors, including diet, geography, host physiology, disease state, and features of the gut itself, contribute to the community composition of the gut microbiota [5]–[9] and can result in discordance with the host phylogeny. Despite the wide variation among individuals, the gut microbiotae of members of the same species are often more similar to one another than to those of other species. But above this level of organization, the composition of these microbial communities is thought to assort according to the broad dietary habits of their hosts [6],[10]. Based on very limited samplings of nonhuman primates, mostly from captive individuals, conspecifics sometimes retain very similar microbial communities (e.g., Hymadryas baboons), but sometimes do not (e.g., western lowland gorillas). And in a previous phylogenetic analysis of mammals based on their gut microbiotae, the great apes were interspersed in multiple clades along with distantly related species [6],[10]. For example, humans, bonobos, and two of the three gorilla species landed in a large “omnivore” clade along with lemurs, an elephant, and an armadillo, whereas the chimpanzees and orangutans grouped with a flying fox in a divergent clade [10]. From such isolated cases of displaced or zoo-raised hosts, it is difficult to extract the degree to which host and environmental factors shape the primate gut microbiota: both factors are certainly important, but their relative contributions cannot be established based on previous sampling.

To address questions pertaining to the stability and variation in the great ape gut microbiota over evolutionary timescales, we performed high-coverage sequencing of the small subunit ribosomal RNA genes [11]–[13] present in the feces of apes collected in their native ranges. The samples from these wild-living hominids included eastern and western lowland gorillas, bonobos, and three subspecies of chimpanzees [14]–[17], as well as two human hosts from different continents. This sampling provided a more comprehensive and less biased view of bacterial species diversity and abundance within the primate distal gut, and revealed that the relationships among microbial communities parallel the host-species phylogeny. Our results indicate that evolutionary changes in host physiology that occurred during the divergence of great apes have been the dominant factor in shaping the distal gut microbial community present in each host species.

## Results

**Figure 1:**
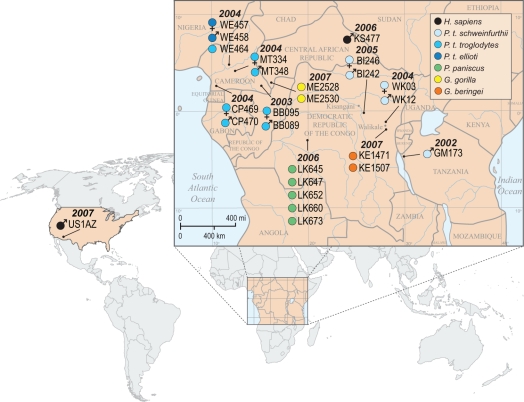
Sample key, locations, collection dates, gender, and taxonomic classification of great apes whose gut microbiotae were analyzed. Note that *P. t. ellioti* is the current nomenclature for *P. t. vellerosus*. Based on mtDNA analyses, each fecal sample represented a different individual.

To investigate factors affecting the diversification of distal gut microbial communities, we assayed the microbiota of humans and multiple members of four other great ape species (eastern lowland gorilla, *Gorilla beringei*; western lowland gorilla, *G. gorilla*; bonobo, *Pan paniscus*; and the three subspecies of chimpanzees; *P. troglodytes troglodytes*, *P. t. schweinfurthii,* and *P. t. ellioti*) sampled in their native ranges (Figure 1). For the 26 great ape samples, we generated a total of 1.5 million reads, of which nearly 1.3 million had a recognizable primer and identifying sequence tag. After quality filtering and length trimming, reads shorter than 150 nucleotides in length were removed, leaving a total of 1,107,714 reads (termed “pyrotags”) that could be assigned taxonomically to a class with greater than 70% bootstrap support. The number of sequencing reads per sample ranged from 14,762 to 178,473, with a median value of 27,945 reads per sample. In addition to characterizing 16S rRNA gene diversity, fecal DNA was assayed for multiple variable regions of ape mitochondrial DNA (mtDNA) (HV1, HV2, HV3, and COIII), and for a Y-chromosome marker, to confirm source species, to determine host gender, and to establish the phylogenetic relationships among hosts. Variation in these host sequences confirmed that each fecal sample was derived from a different individual.

### Phylum-Level Diversity in Gut Microbial Communities

The gut microbiotae of these hosts encompass one archaeal and 18 bacterial phyla, of which five (Actinobacteria, Bacteroidetes, Firmicutes, Proteobacteria, and Verrucomicrobia) were present in all samples. Several phyla not typically observed in gut microbiota of primates, including Euryarchaeota, Acidobacteria, Fibrobacteres, Lentisphaerae, Planctomycetes, and candidate phylum TM7, were recovered at very low relative frequencies (<10^−3^) from at least nine hosts. In addition, five bacterial phyla (Chlamydiae, Chloroflexi, Deferribacteres, OP10, and Gemmatimonadetes) were detected in only one or few hosts. Contributing most to these rare variants was chimpanzee BB089, which had the highest phylum-level diversity of any sample and harbored four of these five uncommon phyla (Table S1).

The three most dominant phyla were Firmicutes, Proteobacteria, and Bacteroidetes, which together constituted over 80% of the reads identified in every sample. Although divergent mammals can harbor broadly similar gut microbiotae at the level of bacterial phylum [7],[10], the two species of gorilla differed from those of other great apes in the relative frequencies of the dominant phyla. Firmicutes was numerically dominant in all great apes but was less common than Proteobacteria and Bacteroidetes in both species of gorilla. Among other phyla represented in all hosts, Actinobacteria was common but only occurred at frequencies of greater than 10% in two of the five bonobos and in a single chimpanzee (CP470), and Verrucomicrobia, usually at frequencies of only 1%–10%, constituted approximately 20% of the microbiota of one human (KS477).

To determine whether great ape species can be distinguished based on the diversity of microbes in their fecal samples, we first performed a phylogenetic analysis of the phylum-level diversity within their gut microbiota. For this analysis, we contructed phylogenetic trees based on the abundance in each sample of pyrotags that were classified to phylum (see Materials and Methods and below for results based on species-level microbial diversity). Despite variation in the distribution and abundance of numerous microbial phyla, these phylum-level phylogenies did not resolve any of the ape species as discrete groups (Figure S1). There were 160 most parsimonious trees, and no clade recovered had greater than 70% bootstrap support. This result is due to both the sporadic occurrence of certain phyla among individual members of the same great ape species and the phylum-level diversity present in the microbiota of chimpanzees, which broadly overlaps that of the other great apes.

### Resolution of Phylotypes and Microbial Species

The majority of the variation in the microbiotae of the great ape hosts is represented as unique pyrotags recovered from a single sample, indicating that differentiation in the gut microbiota among hosts may occur at lower taxonomic levels. Although these 16S rDNA sequences are indicative of a broad range of species-level bacterial diversity in these samples, the source, relevance, and reproducibility of this “rare biosphere” has recently been questioned [18]–[20].

To assess how experimental factors might contribute to the contents of the rare biosphere, we performed a high-coverage technical replicate (WE464R) on an independent preparation of the fecal sample from chimpanzee WE464. Even with sequencing to 3.5 times the depth of the initial sample (51,648 versus 14,762 reads), there were no identical matches for approximately 30% of the reads in the original sample, but when allowing for up to 0.5% sequence divergence between reads (i.e., no more than a single one-nucleotide mismatch or indel), this proportion shrank to less than 10%. Therefore, to assemble the most biologically robust segment of our entire 1,107,714-pyrotag dataset, we grouped sequencing reads by applying a 99.5% identity threshold to correct for most potential sequencing artifacts. We have noted previously that thresholds higher than 97% will inflate richness estimates using amplicon pyrosequencing [20]; however, the approach we take here will be minimally impacted by this artifact. More importantly, application of this high threshold improves the likelihood that clustered pyrotags belong to the same bacterial species, whereas the conventional criterion of 97% sequence identity often unites bacteria typed to different taxonomic groups [21],[22].

**Figure 2:**
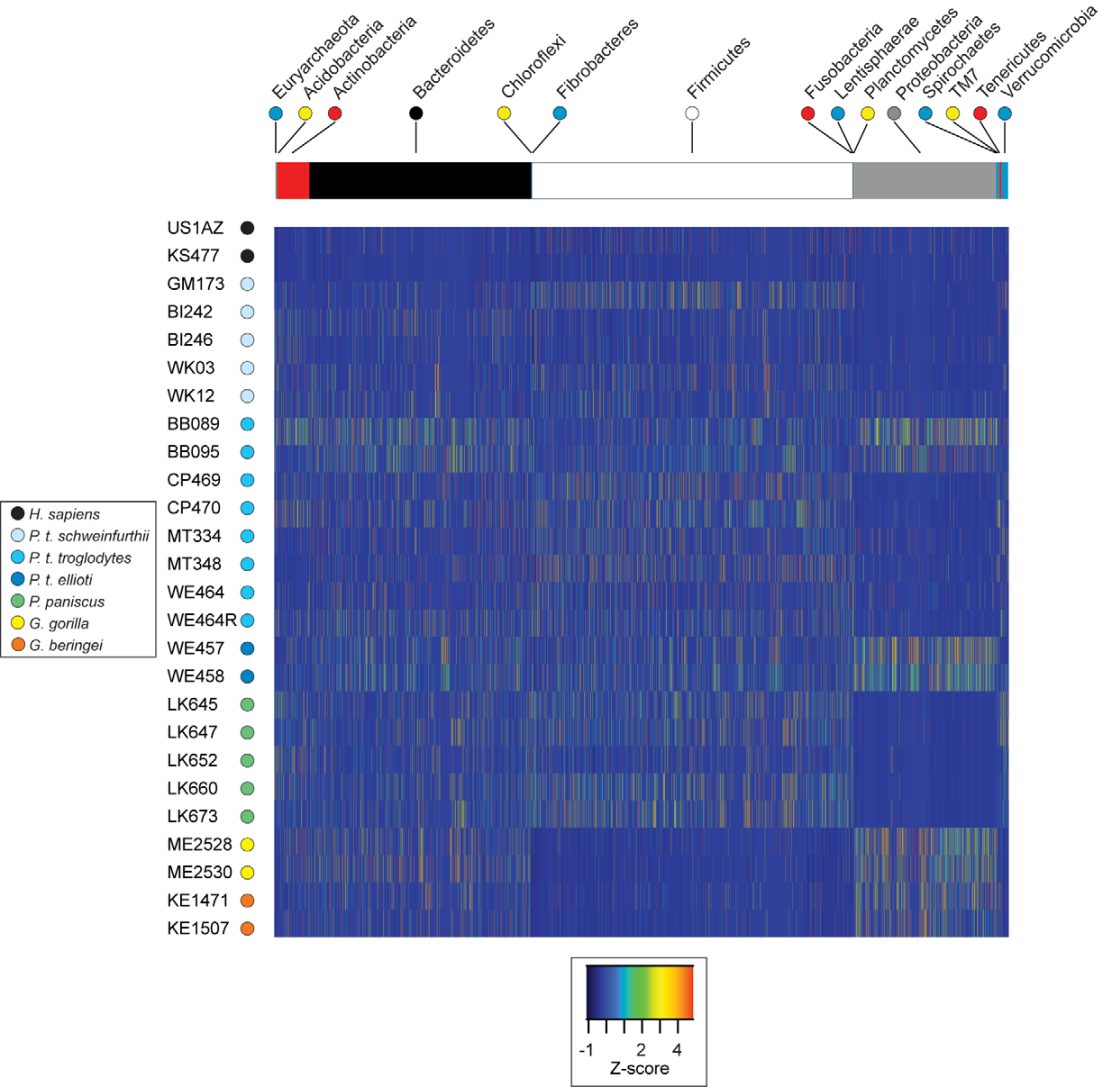
Relative abundance of microbial species across great ape hosts. Rows represent samples (color-coded by host species as in Figure 1); columns represent microbial species (reproducible 99.5% OTUs; *n* = 8,914). Microbial phyla represented in the gut microbiota of these hosts are shown horizontally across the top: OTUs classified to one archaeal (Euryarchaeota) and 14 bacterial phyla, as indicated. Based both on the number of OTUs and on read counts, species classified as Bacteroidetes, Firmicutes, and Proteobacteria are the most dominant phyla in these samples. Samples from four chimpanzees (BB089, BB095, WE457, and WE458) have more proteobacterial reads than do other chimpanzee samples, and samples from both gorilla species contained relatively fewer Firmicutes species than did samples from other ape species. Individual cells are color-coded by *Z*-scores to show the normalized abundance of a particular OTU in one sample relative to the mean abundance across all samples. Intensity of the colors indicates how many standard deviations the observed OTU abundance is above or below the mean.

To determine the degree to which the gut microbial communities present in these great apes are similar in the frequencies of their constituent microbial species, we retained only those 99.5% operational taxonomic units (OTUs) detected in two or more host samples (since unique OTUs are not phylogenetically informative and provide no information about evolutionary relatedness). The set of reproducible 99.5% OTUs contained a total of 1,017,478 reads that formed a total of 8,914 microbial phylotypes (hereafter referred to as “species”), which were used to examine the fine-scale taxonomic structure and similarities of the great ape gut microbiotae (Figure 2).

Whereas our analyses of phylum-level microbial diversity were not sufficiently grained to differentiate great ape species based on their microbiota (a similar limitation encountered by Ley et al. [10] when only 100–200 bacterial sequences were sampled from each host), the assemblage of microbial species (that manifest as reproducible 99.5% OTUs) discriminated the individual primate hosts and assorted them into taxonomic groupings. For example, the two humans shared relatively few phylotypes with other great apes, and both species of gorillas shared high frequencies of proteobacterial phylotypes and very low frequencies of Firmicutes species that were present in the majority of chimpanzee samples. This result indicates that deep sampling of the microbiota is necessary to fully recover the evolutionary signal in gut microbial community data. The number of reproducible microbial species recovered from individual hosts ranged from 265 (in African human KS477) to 3,247 (in chimpanzee WE458, the host for which we obtained the highest number of reads). The highest frequency attained by an individual bacterial species was 27.7% for a phylotype classified as *Megasphaera* (Firmicutes: Clostridium) in the sample from the African human.

### Relatedness of Great Ape Hosts Based on Gut Microbiota

To conduct a phylogenetic analysis of the occurrence and frequencies of species present in the fecal microbial communities in the primate hosts, we treated each microbial species as an individual standard data character assigned to one of six possible states that correspond to order-of-magnitude differences in the normalized frequency of each microbial species in each ape host sample. This approach is similar to that typically used with morphometric data, where, for example, femur length might be treated as a standard character coded with a handful of possible states ranging from very small to very large (e.g., the multi-log-difference size range from mouse to elephant). The aggregate character matrix of several morphometric characters (or, in our case, the frequencies of the various members of a microbial community) can then be analyzed using traditional phylogenetic techniques. The 8,914-species, six-state data matrix was subjected to a heuristic maximum parsimony tree search, as performed previously for the phylum-level tree phylogeny, with 1,000 pseudo-replicates used to assess bootstrap support.

**Figure 3:**
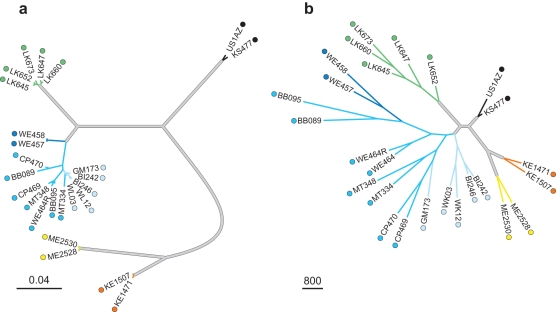
Phylogeny of great apes based on mtDNA sequence variation and composition of host gut microbiota. (A) mtDNA phylogeny. (B) Gut microbiota phylogeny. Color-coding of terminal branches leading to great ape hosts corresponds to those in Figure 1. Internal branches of these trees (thick grey lines), although of different relative lengths, show identical branching orders. In (A), the internal nodes were all supported with bootstrap values greater than 98%; in (B), each internal node showed bootstrap support greater than 98%, except that leading to chimpanzees, which had 68% support.

Because microbial species are defined as reproducible OTUs clustered at 99.5% sequence identity, all 8,914 characters are parsimony-informative, and the phylogenetic analysis recovered a single maximum parsimony tree (*p*-score = 49,475). The unrooted maximum parsimony tree exhibited a species-level topology that was completely congruent with the unrooted mtDNA topology of the hosts (Figure 3). Moreover, these groupings were supported by high bootstrap values (98%–100% for each ape species, with the exception of the chimpanzee clade, which reached only 68% bootstrap support). There are more than 2,000,000 possible unrooted topologies for a ten-taxon phylogenetic tree. Therefore, if one constructs a tree with two humans, two chimpanzees, two bonobos, two eastern lowland gorillas, and two western lowland gorillas, the chance of randomly generating a tree that is entirely congruent with the species tree in placing each species with its conspecific, and also placing the two gorilla species as sister groups and the chimpanzees and bonobos as sister groups (as in Figure 3) is less than 1/2,000,000.

## Discussion

Based on the compositions of the distal gut microbial communities from hosts living in their natural environments, we were able to discriminate species of great apes. The topological concordance between the species-level branching orders obtained for hosts and their microbiotae shows that over evolutionary timescales, host phylogeny is the overriding factor determining the microbial composition of the great ape gut microbiota. This recapitulation of the species relationships in the frequencies of the microbial constituents of their distal gut communities contrasts with previous notions that diet is the most important factor governing the grouping of gut microbiotae within primates [6],[10].

This new view of great ape microbiota evolution emerged as a consequence of the sampling depth, which allowed the recovery of large sets of evolutionarily informative phylotypes. This allowed the application of standard parsimony-based phylogenetic approaches that were based on the frequency of each microbial species shared among hosts. Previous studies of gut microbiotae that surveyed only on the order of 100 sequences per sample [10],[23],[24] could not accurately gauge either the diversity present in complex microbial communities or the relative abundance of the constituent species. Given the species complexity within the distal gut microbiota, it is necessary to obtain more than 10^4^ reads per host to accurately access the relationships among divergent microbial communities. However, recent advances in sequencing methodologies render this number of reads both technically and economically feasible.

The fact that the gut microbial community phylogeny matches the great ape species phylogeny is not readily attributable to factors other than the evolutionary diversification of hosts. For example, the broad geographic range of chimpanzees, as well as the intercontinental distance separating our sampled humans, establishes that geographic proximity is not a major factor in the clustering of microbial communities by host species. Likewise, chimpanzees and gorillas within the same locale exhibited phylogenetically distinct gut microbial communities. That the composition of gut microbiotae assorts to species despite their geographic locations suggests that similarities in local factors, such as those that relate to diet, do not explain the close correspondence between host phylogeny and microbial community composition. To further evaluate whether host species differentiate according to diet, we examined the populations of chloroplast sequences within each fecal sample. Although the diversity of chloroplasts serves as an indicator only of plant diet at the time of sampling, there was no clear indication that the great ape species (except for *G. beringei*) have widely different diets or that the diets of great apes structure according to host phylogeny (Figure S2).

As evident from the differences in relative branch lengths between the mtDNA (Figure 3A) and microbial community (Figure 3B) trees, it is clear that the degree of genetic differentiation between hosts does not fully account for the variation in great ape gut microbiota. The host phylogeny signal that we uncovered can be masked by factors occurring on more proximate timescales (such as diet, geography, or health status). Only by conducting a phylogenetic analysis of communities that have been more deeply sampled is it possible to detect this signal. To assess the degree to which differences in gut microbiota reflect the genetic distance between hosts, we compared the amount of variation assigned to the terminal branches of the tree (i.e., those leading to individual hosts) relative to that encompassed in the seven internal branches that differentiate the five great ape species (grey branches in Figure 3). The species-discriminating branches together represent 73% of the total genetic distance present in the mtDNA phylogeny, but only 7% of the total distance in the tree based on microbial communities. This contrasts with the situation for individual hosts, whose branch lengths together constitute 70% of the distance in the microbial tree but encompass only 11% of the total genetic distance. This disparity reflects the broad variation in microbial communities among members of the same species, as has already been observed in humans [5]–[7],[25]–[28]. Next, to discount the effects of individual variation, we calculated the correlation coefficient between the relative branch lengths of the seven internal branches in the microbial community tree and the corresponding distances in the mtDNA tree. Despite the congruence in branching orders, the branch lengths in the mtDNA tree explain only about 25% of the variation in the microbial community tree. This indicates that gut microbiotae, although diverging in a manner consistent with vertical inheritance, are not changing in a strict time-dependent fashion that reflects the degree of genetic divergence among hosts. The difference in branch length indicates that individual-level variation in microbial community structure is extensive relative to between-species variation.

Our analysis indicates that host phylogeny has a major role in the diversification of distal gut microbial communities in great apes, a conclusion that can become apparent only when sampling is adequate for robust phylogenetic and evolutionary analyses of microbial species compositions. Numerous studies have applied UniFrac and related approaches to establish the relationships among microbial communities derived from a wide range of hosts and environmental sources [29]–[33]. Despite the highly supported tree that we obtained by parsimony analysis, subjecting our dataset to UniFrac did not recover a tree that matches the host-species phylogeny (Figure S3). Unlike parsimony, UniFrac relies on an input tree to specify the evolutionary relationship among bacterial taxa to infer the similarity among microbial communities. However, for a large dataset with nearly 9,000 characters, ensuring the correct inference of tree topology and branch lengths is difficult. The task of inferring an input tree is all the more problematic because of the relatively short and highly variable sequencing reads that are generated for most metagenomic studies. The quality of multiple sequence alignment, which is critical for inferring the guide tree, is greatly impacted by the limited read length, the level of sequence variation, and the propensity towards indel sequencing errors. This problem was almost entirely eliminated from our parsimony analysis (of species abundance data) by performing multiple sequence alignments on sets of reads assigned to a particular taxonomic class, not the entire dataset. Furthermore, when calculating pair-wise sequence identities among reads typed to the same class, indel sequencing errors present in taxonomically different reads are ignored. Since the V6 region has previously been shown to have low phylogenetic congruency with full-length small subunit ribosomal RNA topologies [34], the described methods based on species abundances and community compositions serve as an alternative and complementary approach for analyzing pyrotag data.

With the availability of methods that allow the scrutiny of microbial diversity and community structure at finer levels, the challenge now is to determine how best to characterize each specific environment in order to extract the relevant biological information about its constituents. In the present study, we found that sampling at levels of greater than 10,000 reads per sample, the application of stringent cutoffs for species identity, and the focus on parsimony-informative characters helped resolve host phylogeny as the major determinant of distal gut microbial communities in great apes.

## Materials and Methods

### Sample Collection and Processing

Ape fecal samples used in this study were selected from an existing bank of previously collected specimens [14]–[17]. All samples except one (GM173) were collected from wild-living, non-habituated apes at remote forest sites in Cameroon, the Central African Republic, and the Democratic Republic of the Congo (DRC). Sample GM173 was obtained from a habituated male chimpanzee (Ch-045) in Gombe National Park, Tanzania (Figure 1). In the field, fecal samples were identified to be of likely chimpanzee, gorilla, or bonobo origin by experienced trackers; however, species and subspecies origins were subsequently confirmed in the laboratory by mtDNA analysis. This genetic analysis revealed a limited number of initially misidentified specimens from other mammal species, including a handful of samples that were of human origin. One such sample (KS477) from an unknown individual in the DRC was included in this study. In addition, a fecal sample was supplied by a human male residing in Tucson, Arizona (United States). All fecal samples were collected, stored, and shipped in RNAlater (Ambion). Time, date, and collection site were recorded for each sample. Samples were shipped at ambient temperatures but subsequently stored at −80°C.

DNA was extracted from 200-µl aliquots of thawed fecal samples by spin-column filtration using the QIAamp DNA Stool Kit (Qiagen), following the manufacturer's protocol for isolating DNA for pathogen detection. DNA was quantified on a Qubit fluorometer (Invitrogen) and subjected to PCR amplification of the 16S rDNA region spanned by primers 926F (5′-aaactYaaaKgaattgacgg-3′) and 1492R (5′-tacggYtaccttgttacgactt-3′). Amplicons encompassed the V6 region, which was selected because of prior use and high level of variability [13],[20],[34]–[36]. To multiplex amplicons for inclusion on a single sequencing run (454 Life Sciences/Roche), the appropriate 454 Life Sciences adaptor sequence and a unique three- or four-nucleotide sequence tag (barcode) were added to the 5′ end of the forward and reverse 16S amplification primers. For each primer pair, PCR was performed in triplicate and pooled to minimize PCR biases that might occur in individual reactions. Each 50-µl reaction consisted of 1.25 units of Taq (GE Healthcare), 5 µl of supplied 10× buffer, 0.25 µl of 10 mM dNTP mix (MBI Fermentas), 1.5 µl of 10 mg/ml BSA (New England Biolabs), 0.5 µl of each 10 µM primer, and 40 ng of template DNA, and proceeded at 95°C for 3 min; followed by 25 cycles of 95°C for 30 s, 55°C for 45 s, and 72°C for 90 s; followed by a final extension at 72°C for 10 min. Amplification products were purified on MinElute PCR columns (Qiagen) and quantified. To obtain a similar number of reads from each sample, amplicons were mixed in equal concentrations prior to pyroseqencing. Emulsion PCR and sequencing were performed using a GS-FLX emPCR amplicon kit (454 Life Sciences/Roche), following the manufacturer's protocols. Pyrosequencing proceeded from the barcode at the 5′ end of the 926F primer.

To confirm species and differentiate individual hosts, DNA samples were also tested with primers designed to amplify three hypervariable regions of the D-loop of the great ape mitochondrial genome: HV1 (nucleotides 15997 to 16498), and HV2 and HV3 (nucleotides 16517 to 607). Amplified PCR products were treated with exonuclease I and calf intestinal phosphatase, and directly sequenced from both ends using the amplification primers on an ABI 3700 sequencer (Applied Biosystems). Sequences were assembled in Sequencher (Gene Codes Corporation) and compared to the published mtDNA sequences of great apes. In addition, we used unambiguous polymorphisms to confirm that each sample came from a different individual.

### Informatic Analyses, Quality Filtering, and Taxonomic Assignment

Pyrosequencing flowgrams were converted to sequence reads using software provided by 454 Life Sciences/Roche. Reads were end-trimmed with LUCY [37], using an accuracy threshold of 0.5% per base error probability. Reads lacking exact matches to a recognizable barcode and primer sequence were removed from the dataset, leaving a total of 1,292,542 reads (elsewhere referred to as “pyrotags”) out of the original total of 1,501,806 reads. Reads were assigned to individual samples based on identifying barcode sequences. Barcode and primer sequences were removed from the 5′ end of each read, and the taxonomic origin of each read was established using RDP Classifier. For quality filtering, we excluded the reads that (i) were shorter than 150 nucleotides in length, (ii) received bootstrap support for class assignment lower than 70% (based on RDP Classifier), (iii) mapped to the incorrect region of 16S gene, or (iv) were of chloroplast origin (based on RDP Classifier). This final filter removed more than 99% of the Cyanobacteria reads, leaving only one read that was assigned to genus GpVII.

For sequences classified as Archaea, we required reads to start at position 844–850 (relative to the reference sequence from RDP Aligner); for sequences classified as Bacteria, we required the reads to start at position 851–857. For the 1,107,714 pyrotags that could be assigned to a taxonomic class, we performed all pair-wise comparisons to identify unique sequence types. If two otherwise identical reads differed in length, they were trimmed to the same length. We used RDP Aligner to perform multiple sequence alignments of all unique sequence types. Based on these alignments, we calculated the percent identity of all pairs typed to the same class. Terminal gaps in the 5′ or 3′ end of the alignment were excluded when calculating percent identities.

The clustering step was done using the MCL (Markov Clustering) algorithm with the inflation value set to 1.5 [38]. 99.5% OTUs were partitioned into two sets: unique (present in one sample) and shared (present in at least two samples). Frequencies of shared OTUs were visualized via heatmaps generated in R and used for subsequent analyses.

### Community Relatedness and Phylogenetic Analyses

To establish the degree to which the gut microbiotae of samples were similar with respect to the compositions of their constituent microbes, we constructed phylum-level and species-level phylogenies of hosts based on the frequencies of taxonomically assigned OTUs in their gut microbial communities. Character matrices based on all reads were converted to phylogenetic trees using a parsimony-based approach. Each character corresponds to a taxonomically assigned OTU whose frequency in each sample has been normalized by coding with one of six ordered states reflecting log-unit differences in its occurrence, with a OTU absent from a sample coded as state 0. Given the range in the occurrence of each OTU across samples (from 0 to 83,840 at the phylum level), this resulted in six-state data matrix, which was then subjected to a heuristic maximum parsimony tree search using PAUP version 4.0b10, using default settings. Characters were considered to be ordered, such that transitions between distant states (i.e., samples having very divergent frequencies of a particular phylotype) were more costly than between similar states.

To produce the tree for input to UniFrac, we took the longest read within each OTU as the representative for multiple sequence alignment and generated alignments using RDP Aligner (applying the Bacteria model since only ten of the 8,914 OTUs represented Archaea). The resulting alignment contained 554 aligned nucleotide sites, and the tree relating the 8,914 OTUs was inferred in FastTree version 2.1.1 [39],[40]. Tree topology and sample information were uploaded to the Fast UniFrac Web server [41] for the clustering analysis, using the weighted and normalized options to account for differences in OTU abundance and read depth among samples.

## Supporting Information

Figure S1 **Phylogeny of great apes based on phylum-level classification of gut microbiota.** Color-coding and sample names of individual great ape hosts follow those presented in Figure 1. This phylogeny is based on a character matrix in which each character is a microbial phylum obtained from all classifiable sequencing reads. Each character is coded with one of six ordered states reflecting log-unit differences in the normalized frequency of that particular phylum in each sample. From a total of 160 most parsimonious trees, no clade showed bootstrap support greater than 70%.
(0.26 MB EPS)

Figure S2 **Great ape phylogeny based on diet composition.** Color-coding and sample names of individual great ape hosts correspond to those in Figure 1. Presented is one of 59 most parsimonious trees recovered from analysis of a character matrix encompassing the frequency of 99.5% OTUs of chloroplast reads from each fecal sample (i.e., applying the same approach in the microbial community structure analysis, but instead considering a “snapshot” of the plant component of the recent diet of each animal.) Nodes present in greater than 75% of the 59 most parsimonious trees are indicated with a star. Aside from one species of gorilla, there is no clear indication (at least from this admittedly crude proxy of diet) that the great ape species are distinct, let alone that the deeper structure of the host phylogeny is matched. In other words, the diets of these animals do not appear to be structured simply according to host phylogeny. Accordingly, diet on its own seems an unlikely explanation for the observation of congruence between the microbial community structure tree and the species-level host (mtDNA) phylogeny.
(0.27 MB EPS)

Figure S3 **UniFrac analysis of the microbial communities within the distal gut of great apes.** Color-coding and sample names of great ape hosts correspond to those presented in Figure 1.
(0.14 MB PDF)

Table S1 **Phylum-level prokaryotic diversity in great ape fecal samples.**
(0.05 MB PDF)

## Abbreviations

DRC: Democratic Republic of the Congo
mtDNA: mitochondrial DNA
OTU: operational taxonomic unit
