# Supplementary figures and images for: Evolutionary Relationships of Wild Hominids Recapitulated by Gut Microbial Communities
Source: PLoS Biol. 2010 Nov 16;8(11):e1000546. doi: 10.1371/journal.pbio.1000546 (PMC2982803; doi:10.1371/journal.pbio.1000546)

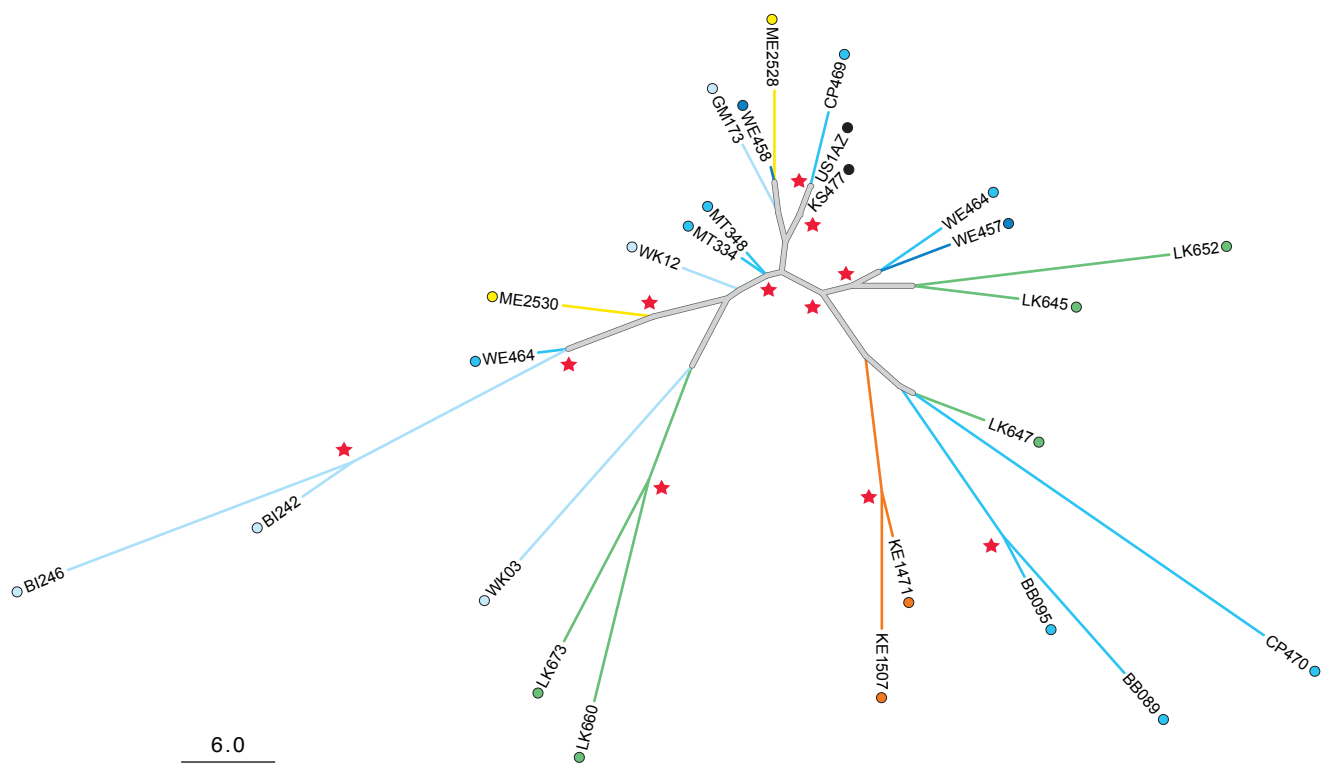

Supplement: Figure S3 — UniFrac analysis of the microbial communities within the distal gut of great apes. Color-coding and sample names of great ape hosts correspond to those presented in Figure 1. (0.14 MB PDF) [file pbio.1000546.s003.pdf]
