# Supplementary material for: Evolutionary Relationships of Wild Hominids Recapitulated by Gut Microbial Communities
Source: PLoS Biol. 2010 Nov 16;8(11):e1000546. doi: 10.1371/journal.pbio.1000546 (PMC2982803; doi:10.1371/journal.pbio.1000546)
Supplement: Table S1 — Phylum-level prokaryotic diversity in great ape fecal samples. (0.05 MB PDF) [file pbio.1000546.s004.pdf]

Table S1. Phylum-level Prokaryotic Diversity in Great Ape fecal samples.

	**Sample** (see figure 1)				
	**US1AZ**	**KS477**	**GM173**	**BI242**	**BI246**
**Phylum**					
A_Euryarchaeota	0 (0.0E+0)	0 (0.0E+0)	5 (9.8E-5)	18 (7.7E-4)	14 (8.5E-4)
B_Acidobacteria	0 (0.0E+0)	0 (0.0E+0)	0 (0.0E+0)	0 (0.0E+0)	0 (0.0E+0)
B_Actinobacteria	944 (5.1E-2)	55 (2.3E-3)	2346 (4.6E-2)	921 (4.0E-2)	783 (4.7E-2)
B_Bacteroidetes	1595 (8.6E-2)	2077 (8.8E-2)	15130 (3.0E-1)	8226 (3.5E-1)	1929 (1.2E-1)
B_Chlamydiae	0 (0.0E+0)	0 (0.0E+0)	0 (0.0E+0)	0 (0.0E+0)	0 (0.0E+0)
B_Chloroflexi	0 (0.0E+0)	0 (0.0E+0)	2 (3.9E-5)	0 (0.0E+0)	0 (0.0E+0)
B_Deferribacteres	0 (0.0E+0)	0 (0.0E+0)	1 (2.0E-5)	0 (0.0E+0)	0 (0.0E+0)
B_Fibrobacteres	0 (0.0E+0)	0 (0.0E+0)	67 (1.3E-3)	0 (0.0E+0)	0 (0.0E+0)
B_Firmicutes	11106 (6.0E-1)	15812 (6.7E-1)	32018 (6.3E-1)	11472 (4.9E-1)	13371 (8.1E-1)
B_Fusobacteria	0 (0.0E+0)	0 (0.0E+0)	0 (0.0E+0)	0 (0.0E+0)	0 (0.0E+0)
B_Gemmatimonadetes	0 (0.0E+0)	0 (0.0E+0)	0 (0.0E+0)	0 (0.0E+0)	0 (0.0E+0)
B_Lentisphaerae	1 (5.4E-5)	0 (0.0E+0)	46 (9.0E-4)	0 (0.0E+0)	0 (0.0E+0)
B_OP10	0 (0.0E+0)	0 (0.0E+0)	0 (0.0E+0)	0 (0.0E+0)	0 (0.0E+0)
B_Planctomycetes	2 (1.1E-4)	0 (0.0E+0)	79 (1.6E-3)	0 (0.0E+0)	0 (0.0E+0)
B_Proteobacteria	4826 (2.6E-1)	1068 (4.5E-2)	237 (4.7E-3)	518 (2.2E-2)	387 (2.3E-2)
B_Spirochaetes	2 (1.1E-4)	0 (0.0E+0)	145 (2.9E-3)	2047 (8.8E-2)	39 (2.4E-3)
B_TM7	0 (0.0E+0)	0 (0.0E+0)	0 (0.0E+0)	2 (8.6E-5)	1 (6.0E-5)
B_Tenericutes	11 (5.9E-4)	0 (0.0E+0)	33 (6.5E-4)	29 (1.2E-3)	0 (0.0E+0)
B_Verrucomicrobia	4 (2.2E-4)	4656 (2.0E-1)	723 (1.4E-2)	6 (2.6E-4)	15 (9.1E-4)
Total reads	18491	23668	50832	23239	16539

Cells contain numbers of classifiable reads (and their frequencies) for a given sample.

Table S1. Phylum-level Prokaryotic Diversity in Great Ape fecal samples.

	**Sample** (see figure 1)				
	**WK3**	**WK12**	**BB089**	**BB095**	**MT334**
**Phylum**					
A_Euryarchaeota	22	0	16	18	3
	(1.2E-3)	(0.0E+0)	(1.8E-4)	(2.1E-4)	(1.1E-4)
B_Acidobacteria	4	1	9	36	1
	(2.2E-4)	(4.2E-5)	(1.0E-4)	(4.3E-4)	(3.8E-5)
B_Actinobacteria	952	759	6183	1481	502
	(5.1E-2)	(3.2E-2)	(7.0E-2)	(1.8E-2)	(1.9E-2)
B_Bacteroidetes	4236	10051	32019	26837	7505
	(2.3E-1)	(4.3E-1)	(3.7E-1)	(3.2E-1)	(2.8E-1)
B_Chlamydiae	0	0	1	0	0
	(0.0E+0)	(0.0E+0)	(1.1E-5)	(0.0E+0)	(0.0E+0)
B_Chloroflexi	0	0	2	0	0
	(0.0E+0)	(0.0E+0)	(2.3E-5)	(0.0E+0)	(0.0E+0)
B_Deferribacteres	0	0	0	0	0
	(0.0E+0)	(0.0E+0)	(0.0E+0)	(0.0E+0)	(0.0E+0)
B_Fibrobacteres	0	0	50	377	20
	(0.0E+0)	(0.0E+0)	(5.7E-4)	(4.5E-3)	(7.5E-4)
B_Firmicutes	12902	10663	20182	27154	12521
	(7.0E-1)	(4.5E-1)	(2.3E-1)	(3.2E-1)	(4.7E-1)
B_Fusobacteria	0	0	0	0	0
	(0.0E+0)	(0.0E+0)	(0.0E+0)	(0.0E+0)	(0.0E+0)
B_Gemmatimonadetes	0	0	2	0	0
	(0.0E+0)	(0.0E+0)	(2.3E-5)	(0.0E+0)	(0.0E+0)
B_Lentisphaerae	0	0	1	0	8
	(0.0E+0)	(0.0E+0)	(1.1E-5)	(0.0E+0)	(3.0E-4)
B_OP10	0	0	22	0	0
	(0.0E+0)	(0.0E+0)	(2.5E-4)	(0.0E+0)	(0.0E+0)
B_Planctomycetes	0	0	12	3	15
	(0.0E+0)	(0.0E+0)	(1.4E-4)	(3.6E-5)	(5.7E-4)
B_Proteobacteria	242	1954	28818	28030	5567
	(1.3E-2)	(8.3E-2)	(3.3E-1)	(3.3E-1)	(2.1E-1)
B_Spirochaetes	3	0	59	172	62
	(1.6E-4)	(0.0E+0)	(6.7E-4)	(2.0E-3)	(2.3E-3)
B_TM7	0	0	21	5	0
	(0.0E+0)	(0.0E+0)	(2.4E-4)	(5.9E-5)	(0.0E+0)
B_Tenericutes	70	85	6	50	71
	(3.8E-3)	(3.6E-3)	(6.8E-5)	(5.9E-4)	(2.7E-3)
B_Verrucomicrobia	62	19	301	81	251
	(3.4E-3)	(8.1E-4)	(3.4E-3)	(9.6E-4)	(9.5E-3)
Total reads	18493	23532	87704	84244	26526

Cells contain numbers of classifiable reads (and their frequencies) for a given sample.

Table S1. Phylum-level Prokaryotic Diversity in Great Ape fecal samples.

	**Sample** (see figure 1)				
**Phylum**	**MT348**	**WE464**	**CP469**	**CP470**	**WE457**
A_Euryarchaeota	2<br>(5.6E-5)	3<br>(2.0E-4)	0<br>(0.0E+0)	11<br>(1.2E-4)	30<br>(4.2E-4)
B_Acidobacteria	3<br>(8.4E-5)	6<br>(4.1E-4)	0<br>(0.0E+0)	8<br>(8.5E-5)	10<br>(1.4E-4)
B_Actinobacteria	126<br>(3.5E-3)	192<br>(1.3E-2)	845<br>(3.0E-2)	12632<br>(1.3E-1)	387<br>(5.4E-3)
B_Bacteroidetes	7612<br>(2.1E-1)	4219<br>(2.9E-1)	4842<br>(1.7E-1)	28857<br>(3.1E-1)	13861<br>(1.9E-1)
B_Chlamydiae	0<br>(0.0E+0)	0<br>(0.0E+0)	0<br>(0.0E+0)	0<br>(0.0E+0)	0<br>(0.0E+0)
B_Chloroflexi	1<br>(2.8E-5)	0<br>(0.0E+0)	0<br>(0.0E+0)	22<br>(2.3E-4)	0<br>(0.0E+0)
B_Deferribacteres	0<br>(0.0E+0)	0<br>(0.0E+0)	0<br>(0.0E+0)	1<br>(1.1E-5)	1<br>(1.4E-5)
B_Fibrobacteres	43<br>(1.2E-3)	0<br>(0.0E+0)	15<br>(5.4E-4)	84<br>(8.9E-4)	11<br>(1.5E-4)
B_Firmicutes	26201<br>(7.3E-1)	9054<br>(6.1E-1)	21441<br>(7.7E-1)	49326<br>(5.2E-1)	16027<br>(2.2E-1)
B_Fusobacteria	0<br>(0.0E+0)	56<br>(3.8E-3)	0<br>(0.0E+0)	0<br>(0.0E+0)	0<br>(0.0E+0)
B_Gemmatimonadetes	0<br>(0.0E+0)	0<br>(0.0E+0)	0<br>(0.0E+0)	0<br>(0.0E+0)	0<br>(0.0E+0)
B_Lentisphaerae	212<br>(5.9E-3)	0<br>(0.0E+0)	31<br>(1.1E-3)	193<br>(2.0E-3)	1<br>(1.4E-5)
B_OP10	0<br>(0.0E+0)	0<br>(0.0E+0)	0<br>(0.0E+0)	0<br>(0.0E+0)	0<br>(0.0E+0)
B_Planctomycetes	48<br>(1.3E-3)	2<br>(1.4E-4)	0<br>(0.0E+0)	0<br>(0.0E+0)	0<br>(0.0E+0)
B_Proteobacteria	768<br>(2.1E-2)	1006<br>(6.8E-2)	192<br>(6.9E-3)	593<br>(6.3E-3)	41728<br>(5.8E-1)
B_Spirochaetes	830<br>(2.3E-2)	1<br>(6.8E-5)	223<br>(8.0E-3)	2177<br>(2.3E-2)	80<br>(1.1E-3)
B_TM7	0<br>(0.0E+0)	0<br>(0.0E+0)	0<br>(0.0E+0)	11<br>(1.2E-4)	0<br>(0.0E+0)
B_Tenericutes	0<br>(0.0E+0)	197<br>(1.3E-2)	52<br>(1.9E-3)	82<br>(8.7E-4)	84<br>(1.2E-3)
B_Verrucomicrobia	55<br>(1.5E-3)	26<br>(1.8E-3)	119<br>(4.3E-3)	332<br>(3.5E-3)	6<br>(8.3E-5)
Total reads	35901	14762	27760	94329	72226

Cells contain numbers of classifiable reads (and their frequencies) for a given sample.

Table S1. Phylum-level Prokaryotic Diversity in Great Ape fecal samples.

	**Sample** (see figure 1)				
	**WE458**	**LK645**	**LK647**	**LK652**	**LK660**
**Phylum**					
A_Euryarchaeota	23	9	7	29	82
	(1.3E-4)	(3.3E-4)	(2.7E-4)	(1.4E-3)	(2.9E-3)
B_Acidobacteria	0	0	2	3	3
	(0.0E+0)	(0.0E+0)	(7.6E-5)	(1.5E-4)	(1.1E-4)
B_Actinobacteria	1805	3021	894	2519	1527
	(1.0E-2)	(1.1E-1)	(3.4E-2)	(1.2E-1)	(5.5E-2)
B_Bacteroidetes	34123	4254	5212	4701	6156
	(1.9E-1)	(1.6E-1)	(2.0E-1)	(2.3E-1)	(2.2E-1)
B_Chlamydiae	0	0	0	0	0
	(0.0E+0)	(0.0E+0)	(0.0E+0)	(0.0E+0)	(0.0E+0)
B_Chloroflexi	0	0	0	1	4
	(0.0E+0)	(0.0E+0)	(0.0E+0)	(4.9E-5)	(1.4E-4)
B_Deferribacteres	0	0	0	0	0
	(0.0E+0)	(0.0E+0)	(0.0E+0)	(0.0E+0)	(0.0E+0)
B_Fibrobacteres	0	170	0	66	154
	(0.0E+0)	(6.2E-3)	(0.0E+0)	(3.2E-3)	(5.5E-3)
B_Firmicutes	56825	19024	18879	12944	19585
	(3.2E-1)	(7.0E-1)	(7.2E-1)	(6.3E-1)	(7.0E-1)
B_Fusobacteria	3	0	0	0	0
	(1.7E-5)	(0.0E+0)	(0.0E+0)	(0.0E+0)	(0.0E+0)
B_Gemmatimonadetes	2	0	0	0	0
	(1.1E-5)	(0.0E+0)	(0.0E+0)	(0.0E+0)	(0.0E+0)
B_Lentisphaerae	8	1	0	15	9
	(4.5E-5)	(3.7E-5)	(0.0E+0)	(7.3E-4)	(3.2E-4)
B_OP10	0	0	0	0	0
	(0.0E+0)	(0.0E+0)	(0.0E+0)	(0.0E+0)	(0.0E+0)
B_Planctomycetes	1	0	0	0	0
	(5.6E-6)	(0.0E+0)	(0.0E+0)	(0.0E+0)	(0.0E+0)
B_Proteobacteria	83840	195	732	67	158
	(4.7E-1)	(7.1E-3)	(2.8E-2)	(3.3E-3)	(5.7E-3)
B_Spirochaetes	50	236	117	40	80
	(2.8E-4)	(8.6E-3)	(4.5E-3)	(1.9E-3)	(2.9E-3)
B_TM7	3	0	0	1	7
	(1.7E-5)	(0.0E+0)	(0.0E+0)	(4.9E-5)	(2.5E-4)
B_Tenericutes	1430	130	166	74	77
	(8.0E-3)	(4.8E-3)	(6.3E-3)	(3.6E-3)	(2.8E-3)
B_Verrucomicrobia	360	290	251	79	93
	(2.0E-3)	(1.1E-2)	(9.6E-3)	(3.8E-3)	(3.3E-3)
Total reads	178473	27330	26260	20539	27935

Cells contain numbers of classifiable reads (and their frequencies) for a given sample.

Table S1. Phylum-level Prokaryotic Diversity in Great Ape fecal samples.

	**Sample** (see figure 1)				
**Phylum**	**LK673**	**ME2528**	**ME2530**	**KE1471**	**KE1507**
A_Euryarchaeota	15	0	0	5	1
	(5.4E-4)	(0.0E+0)	(0.0E+0)	(1.3E-4)	(3.5E-5)
B_Acidobacteria	2	1	1	1	1
	(7.2E-5)	(3.2E-5)	(3.4E-5)	(2.5E-5)	(3.5E-5)
B_Actinobacteria	741	803	411	510	1318
	(2.7E-2)	(2.6E-2)	(1.4E-2)	(1.3E-2)	(4.6E-2)
B_Bacteroidetes	4306	11810	15352	13176	10578
	(1.5E-1)	(3.8E-1)	(5.2E-1)	(3.3E-1)	(3.7E-1)
B_Chlamydiae	0	0	0	0	0
	(0.0E+0)	(0.0E+0)	(0.0E+0)	(0.0E+0)	(0.0E+0)
B_Chloroflexi	5	1	0	2	0
	(1.8E-4)	(3.2E-5)	(0.0E+0)	(5.0E-5)	(0.0E+0)
B_Deferribacteres	0	0	0	0	0
	(0.0E+0)	(0.0E+0)	(0.0E+0)	(0.0E+0)	(0.0E+0)
B_Fibrobacteres	81	0	1	1	2
	(2.9E-3)	(0.0E+0)	(3.4E-5)	(2.5E-5)	(7.0E-5)
B_Firmicutes	22313	2092	954	4439	2506
	(8.0E-1)	(6.7E-2)	(3.3E-2)	(1.1E-1)	(8.8E-2)
B_Fusobacteria	0	1	0	1	0
	(0.0E+0)	(3.2E-5)	(0.0E+0)	(2.5E-5)	(0.0E+0)
B_Gemmatimonadetes	0	0	0	0	0
	(0.0E+0)	(0.0E+0)	(0.0E+0)	(0.0E+0)	(0.0E+0)
B_Lentisphaerae	49	0	1	0	0
	(1.8E-3)	(0.0E+0)	(3.4E-5)	(0.0E+0)	(0.0E+0)
B_OP10	0	0	1	0	0
	(0.0E+0)	(0.0E+0)	(3.4E-5)	(0.0E+0)	(0.0E+0)
B_Planctomycetes	0	0	9	1	1
	(0.0E+0)	(0.0E+0)	(3.1E-4)	(2.5E-5)	(3.5E-5)
B_Proteobacteria	230	16694	12554	21648	14174
	(8.2E-3)	(5.3E-1)	(4.3E-1)	(5.4E-1)	(5.0E-1)
B_Spirochaetes	56	3	6	108	15
	(2.0E-3)	(9.5E-5)	(2.0E-4)	(2.7E-3)	(5.2E-4)
B_TM7	1	1	0	2	0
	(3.6E-5)	(3.2E-5)	(0.0E+0)	(5.0E-5)	(0.0E+0)
B_Tenericutes	36	12	5	66	13
	(1.3E-3)	(3.8E-4)	(1.7E-4)	(1.7E-3)	(4.5E-4)
B_Verrucomicrobia	120	13	8	16	9
	(4.3E-3)	(4.1E-4)	(2.7E-4)	(4.0E-4)	(3.1E-4)
Total reads	27955	31431	29303	39976	28618

Cells contain numbers of classifiable reads (and their frequencies) for a given sample.
